# Passive immunization with Pneumovax^®^ 23 and pneumolysin in combination with vancomycin for pneumococcal endophthalmitis

**DOI:** 10.1186/1471-2415-13-8

**Published:** 2013-03-11

**Authors:** Melissa E Sanders, Sidney Taylor, Nathan Tullos, Erin W Norcross, Quincy C Moore, Hilary Thompson, Lauren B King, Mary E Marquart

**Affiliations:** 1 Department of Microbiology, University of Mississippi Medical Center, Jackson, MS 39216, USA; 2 Department of Biometry, Louisiana State University Health Sciences Center School of Public Health, New Orleans, LA 70112, USA

**Keywords:** *Streptococcus pneumoniae*, Bacterial endophthalmitis, Capsule, Pneumolysin, Vaccination

## Abstract

**Background:**

Capsule and pneumolysin (PLY) are two major virulence factors of *Streptococcus pneumoniae*. *S. pneumoniae* is one of the leading causes of bacterial endophthalmitis. The aim of this study is to determine whether passive immunization with the 23-valent pneumococcal polysaccharide vaccine (Pneumovax^®^ 23; PPSV23) or PLY protects against pneumococcal endophthalmitis.

**Methods:**

New Zealand white rabbits were passively immunized with antiserum to PLY, PPSV23, a mixture of PPSV23/PLY, or PBS (mock). Vitreous was infected with a clinical strain of *S. pneumoniae*. In a separate group of experiments, vancomycin was injected 4 hours post-infection (PI) for each passively immunized group. Severity of infection, bacterial recovery, myeloperoxidase (MPO) activity and percent loss of retinal function were determined.

**Results:**

Passive immunization with each antiserum significantly lowered clinical severity compared to mock immunization (PPSV23 = 9.19, PPSV23/PLY = 10.45, PLY = 8.71, Mock = 16.83; P = 0.0467). A significantly higher bacterial load was recovered from the vitreous of PLY passively immunized rabbits 24 hours PI (7.87 log_10_ CFU) compared to controls (7.10 log_10_ CFU; P = 0.0134). Retinas from immunized rabbits were more intact. Vitreous of PLY (2.88 MPO untis/mL) and PPSV23/PLY (2.17) passively immunized rabbits had less MPO activity compared to controls (5.64; P = 0.0480), and both passive immunizations (PLY = 31.34% loss of retinal function, PPSV23/PLY = 27.44%) helped to significantly preserve retinal function compared to controls (64.58%; P = 0.0323). When vancomycin was administered 4 hours PI, all eyes were sterile at 24 hours PI. A significantly lower clinical severity was observed for rabbits administered the combination immunization (5.29) or PPSV23 (5.29) with vancomycin treatment compared to controls (17.68; P = 0.0469).

**Conclusions:**

Passive immunization with antisera to these antigens is effective in reducing clinical severity of pneumococcal endophthalmitis in rabbits. Addition of vancomycin to immunization is effective at eliminating the bacteria.

## Background

*Streptococcus pneumoniae* (pneumococcus) is a gram-positive coccus that is a normal resident of the human nasopharynx. This bacterium is an opportunistic cause of many human diseases such as bacteremia, meningitis, otitis media, peritonitis, pneumonia, and sinusitis [1-4]. Likewise, *S. pneumoniae* has been reported to be a common cause of bacterial endophthalmitis, often as a result of ocular surgery complications [5-7]. One study of note reported that out of 497 cases of bacterial endophthalmitis at the Wills Eye Institute from 1989–2000, *S. pneumoniae* was one of the top 3 causes of infection in cases that caused pathology in the first 3 days [8]. Intraocular infection with this bacterium often leads to blindness or loss of the eye [7,9,10].

One of the major virulence factors of *S. pneumoniae* is the polysaccharide capsule [11-16]. There are at least 91 different capsule types of *S. pneumoniae*, and purified capsules from several types have been used as immunogens in vaccines for protection against invasive pneumococcal diseases. Two pneumococcal vaccines are currently licensed for use in the United States and are based on the polysaccharide capsule, the 13-valent conjugate vaccine (Prevnar 13^®^; Pfizer (formerly Wyeth Pharmaceuticals), New York, NY, USA) and the 23-valent polysaccharide vaccine (Pneumovax^®^ 23; PPSV23; Merck, Whitehouse Station, NJ, USA) [17-19].

Pneumovax^®^ 23 contains purified polysaccharides of the 23 most commonly found serotypes in pneumococcal infections (1, 2, 3, 4, 5, 6B, 7 F, 8, 9 N, 9 V, 10A, 11A, 12 F, 14, 15B, 17 F, 18C, 19 F, 19A, 20, 22 F, 23 F, and 33 F; listed on the drug literature insert). In studies thus far, immune protection has been tested for non-ocular diseases. A study by Jeurissen et al. [20] elucidated the immune response to the polysaccharides allows protection from certain diseases. Robbins et al. [18] observed 90% protection from blood isolates and 85% protection from colonization of normally sterile sites by *S. pneumoniae* with these serotypes. Regarding ocular studies, this laboratory previously showed that capsule is important for pneumococcal endophthalmitis [21]. The pathogenesis of a parent clinical isolate and isogenic capsule mutant were compared in a rabbit endophthalmitis model. Capsule was determined to be important for infection and retinal damage in this model. These findings led to the question of whether PPSV23 could protect the eye from damage observed during pneumococcal endophthalmitis.

Another virulence factor of *S. pneumoniae* is pneumolysin (PLY), a cholesterol-dependent cytolysin [22]. PLY binds to cholesterol in the membranes of host cells and forms pores, often resulting in cell lysis [23-25]. Pure toxin injected into the vitreous of rabbits shows the same severity of infection as living pneumococcus injected into the vitreous [26]. PLY mutants of *S. pneumoniae* cause less severe pathology than their parent strains in rabbit models [26,27]. A previous study by this laboratory showed that vaccination with PLY significantly lowered pathogenesis caused by pneumococcal endophthalmitis in a rabbit model at 24 and 48 hours PI. Vaccination with PLY also significantly lowered retinal damage at 48 hours post-infection (PI) [28]. However, the bacterial load was high in the vitreous and some (albeit much less) ocular damage still occurred in the immunized animals, which prompted further investigation. Due to the previous findings that both capsule and PLY are important in the pathogenesis of pneumococcal endophthalmitis, the current study was undertaken to determine whether antisera specific for pneumococcal capsule, alone or in combination with PLY-antisera, could provide protection against this disease.

## Methods

### Model

Specific pathogen-free (SPF) New Zealand white rabbits (Harlan Sprague Dawley, Inc, Oxford, Michigan, USA) were used in these studies and maintained according to the ARVO Statement for the Use of Animals in Ophthalmic and Vision Research and the Institutional Animal Care and Use Committee of the University of Mississippi Medical Center.

### Antiserum preparation

Antisera were produced by active primary immunization of rabbits as previously described [28,29]. The immunogens used in this study were PPSV23, PPSV23/ΨPLY (a form of recombinant PLY with a Trp433Phe substitution that results in retention of only 1% cytolytic activity [30]), or PBS (“mock”). We previously reported the effect of active immunization with ΨPLY and vitreal challenge with a clinical ocular strain of *S. pneumoniae*[28]; therefore, this experimental group (ΨPLY active immunization) was not included in the current study. Each rabbit was bled before the first immunization and one week after each boost for the isolation of serum.

Serum anti-PPSV23, anti-PLY, and anti-19 F IgG titers were determined by ELISA [31]. As a control for the possible production of antibodies against the histidine tag of ΨPLY, ELISA plates were coated with an unrelated protein containing the same type of histidine tag to determine IgG titers to the histidine tag in the rabbit serum. Since PPSV23 is contaminated with up to 5% cell wall, serum was adsorbed with purified cell wall polysaccharide before performing the ELISA [32]. Plates were coated with PPSV23 for determination of vaccine-specific IgG titer, ΨPLY for the determination of PLY-specific IgG titer, or 19 F purified polysaccharide to determine the titer specific to the clinical isolate capsule type. Each titer was defined as the highest dilution which was double the background absorbance (A_410_).

### Passive immunization

For passive immunization studies, rabbits were administered 1 mL of antiserum against PPSV23, PPSV23/PLY, PLY, or mock antiserum in the ear vein at the same time as infection. Mock antiserum had no titer. All other sera, after pre-adsorption with cell-wall polysaccharide where applicable, had specific titers ≥ 12,800.

### Bacterial growth

*S. pneumoniae* E335, a serotype 19 F clinical endophthalmitis strain, was provided by Regis Kowalski (Charles T. Campbell Eye Microbiology Lab, Pittsburgh, Pennsylvania, USA) and was grown to approximately 10^8^ colony-forming units (CFU) per mL as previously described [28]. Accuracy of the bacterial CFU was verified by plate counts of serial dilutions.

### Infection

Each rabbit was anesthetized and infected intravitreously with approximately 10^2^ CFU in 10 μL as previously described [28].

### Vancomycin treatment

At 4 hours PI, 100 μL of vitreous was aspirated from passively immunized rabbits using a 30-gauge needle. Each eye was then injected with 100 uL of vancomycin (1 mg/ 0.1 mL; Sigma-Aldrich) using a 30-gauge needle.

### Slit lamp examination (SLE)

SLE for endophthalmitis was previously described [33]. In short, eight parameters were used for determining the severity of endophthalmitis. Each parameter was given a grade from 0 (no pathogenesis) to 4 (maximal pathogenesis), resulting in a total score with a theoretical maximum of 32.

### CFU recovery

Vitreous was removed from each eye at 24 hours PI for all experiments using a 22-gauge needle. The vitreous samples were serially diluted, cultured in triplicate on blood agar, and incubated in 5% CO_2_ at 37°C overnight for quantitation of log_10_ CFU per mL recovered.

### Whole blood survival assay

A bacterial survival assay was performed on blood from the rabbits that were actively immunized to generate the antisera used in the passive immunization experiments using a previously published technique [34]. In short, 1 mL of whole blood from PPSV23, PPSV23/PLY, or mock immunized rabbits was mixed with 100 CFU of *S. pneumoniae* in Todd Hewitt broth containing 0.5% yeast extract (THY). Each sample was placed in a shaker incubator at 37°C for 3 hours, serially diluted, plated in triplicate, and incubated overnight to determine bacterial survival.

### Histopathology

Whole eyes were removed, and histologic sectioning and staining were performed (Excalibur Pathology, Inc., Moore, OK). Hematoxylin and eosin staining was used to stain eosinophils and general ocular architecture.

### Myeloperoxidase (MPO) activity assay

The MPO activity of neutrophils in infected vitreous of passively immunized rabbits was determined using a colorimetric assay as described previously [35]. Uninfected vitreous served as a background control, and purified MPO (Sigma-Aldrich) served as a positive control. MPO activity was expressed as MPO units per mL.

### Vitreal Immunoglobulin G (IgG)

The presence of PPSV23 or PLY specific IgG in the vitreous was determined using ELISA [32]. Samples of vitreous were taken 24 hours PI. ELISA plates were coated with either PPSV23 or PLY. A positive titer was defined as producing at least double the background absorbance (A_410_).

### Electroretinography (ERG)

ERG was used to quantify the retinal response, as previously described [21]. Percent loss of retinal function was calculated as {[1 – (experimental B-wave amplitude / baseline B-wave amplitude)] × 100}. ERG results with no demonstrable waveform were assigned a value of 0.00 μV.

### Statistics

Clinical SLE scores and bacterial CFU data were analyzed as dependent variables using a two level factorial model in the analysis of variance. The main factor was treatment (mock, PPSV23 or PPSV23/PLY). The model was analyzed as a repeated measures model where within subject correlation (eyes within rabbit) was taken into account [36]. The error term was based on two replications of each of the experiments. Mean values for the interaction of time by treatment were separated in post-hoc testing using a method of simulation for alpha level adjustment for multiple comparisons [37]. Whole blood survival data were analyzed using the non-parametric Mann–Whitney *U* test [38]. The alpha level for all hypothesis tests in all statistical procedures was pre-set at 0.05. All data analysis and manipulation was carried out using the Statistical Analysis System (SAS Institute, Cary, NC, USA). All p values stated in results and figures are adjusted p values from post-hoc t tests following a significant overall F test in the ANOVA models.

## Results

### Antibody titers

Antisera to PPSV23 had specific anti-PPSV23 IgG titers ≥ 12,800. PLY-specific serum IgG titers were ≥ 51,200. Antisera to PPSV23/PLY had anti-PPSV23 IgG titers ≥ 12,800 and anti-PLY IgG titers ≥ 51,200. No IgG titer was observed for control (mock) antisera (< 100). A low titer (~ 800) for the histidine tag of the unrelated recombinant protein was observed in antisera to PLY and PPSV23/PLY. Anti-19 F IgG titers were between 6,400 and 12,800 for PPSV23/PLY antisera and 1,600 and 12,800 for PPSV23 antisera.

### Endophthalmitis severity in immunized rabbits

**Figure 1:**
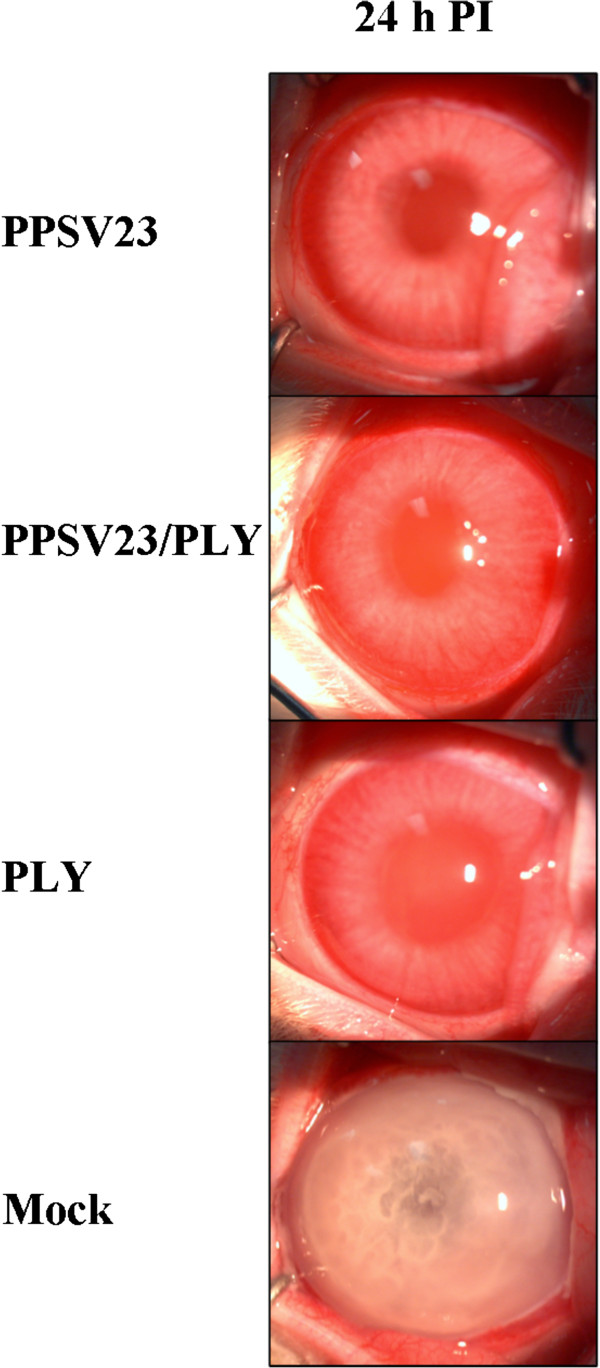
Representative eyes of PPSV23, PPSV23/PLY, PLY, or mock passively immunized rabbits at 24 hours PI.

Passive administration of antisera from all experimental groups was able to reduce SLE scores significantly as compared to rabbits administered mock serum at 24 hours PI (P = 0.0467; Figure 1, Table 1). None of the passive immunization experimental groups (PPSV23, PPSV23/PLY, and PLY antisera) were significantly different from each other in mean SLE scores (P = 0.8846). A more severe infiltration of immune cells was observed in the aqueous humor (Figure 1) and vitreous humor of the mock immunized rabbits as compared to all other passively immunized groups. Passive immunization experiments were terminated at 24 hours PI because the control rabbits were euthanized due to the severity of the infection. When vancomycin was injected into the vitreous at 4 hours PI, a significantly lower severity of infection was observed for PPSV23/PLY passively immunized rabbits at 24 hours PI as compared to PLY and mock passively immunized groups (P = 0.0469; Table 2). The subtle differences between vancomycin-treated eyes of different groups were not able to be distinguished by photography (not shown).

**Table 1:** Mean SLE scores* and percent loss of retinal function* of passively immunized rabbits

	**SLE scores**	**% Loss retinal function**
	**24 hours PI**	**24 hours PI**
PPSV23 Passive	9.19 ± 1.67^+^	40.12 ± 24.26
PPSV23/ΨPLY Passive	10.45 ± 1.67^+^	27.44 ± 16.69^+^
ΨPLY Passive	8.71 ± 1.67^+^	31.34 ± 18.34^+^
Mock Passive	16.83 ± 1.67	64.58 ± 27.15

* ± SEM.

^+^Significantly lower than Mock.

N = 12 eyes per group for SLE and 6 eyes per group for retinal function.

**Table 2:** Mean SLE scores* and percent loss of retinal function* of passively immunized rabbits administered vancomycin 4 hours post-infection

	**SLE scores**	**% Loss retinal function**
	**24 hours PI**	**24 hours PI**
PPSV23 Passive	6.00 ± 0.53	5.12 ± 7.56^+^
PPSV23/ΨPLY Passive	5.09 ± 0.43^#^	5.29 ± 9.62^+^
ΨPLY Passive	6.61 ± 0.23	9.07 ± 15.66
Mock Passive	6.92 ± 0.47	17.68 ± 13.36

* ± SEM.

^#^Significantly lower than PLY and Mock.

^+^Significantly lower than Mock.

N = 4 eyes per group for SLE and 4 eyes per group for retinal function.

### CFU recovery

Rabbits that were administered mock antisera had a significantly lower mean log_10_ CFU/mL recovered from the vitreous than rabbits passively immunized with PLY antisera (P = 0.0134). No significant difference was observed among any other groups tested at 24 hours PI (P = 0.2681; Table 3). All eyes were sterile at 24 hours PI regardless of immunization when vancomycin was injected into the vitreous at 4 hours PI.

**Table 3:** **Log**_**10 **_CFU/mL* recovered from vitreous of passively immunized rabbits

**Group**	**n value**	**Log**_**10 **_**CFU/mL**
		**24 hours PI**
PPSV23	12	7.54 ± 0.17
PPSV23/ΨPLY	12	7.47 ± 0.17
ΨPLY	12	7.87 ± 0.17^+^
Mock	12	7.10 ± 0.17

* ± SEM.

^+^Significantly higher than mock immunized rabbits at same time point.

### Whole blood survival assay

Whole blood from PPSV23 actively immunized rabbits was able to kill bacteria significantly better than whole blood from mock immunized rabbits (15 ± 5 CFU and 1302 ± 248 CFU, respectively; P = 0.0004; n = 9 and 6; respectively). Also, whole blood from PPSV23/PLY actively immunized rabbits was significantly better at killing bacteria than whole blood from mock immunized rabbits (17 ± 7 CFU and 1,499 ± 100 CFU, respectively; P < 0.0001; n = 9 per group). No significant difference was observed between PPSV23 and PPSV23/PLY whole blood (P = 0.8475).

### Histopathology

**Figure 2:**
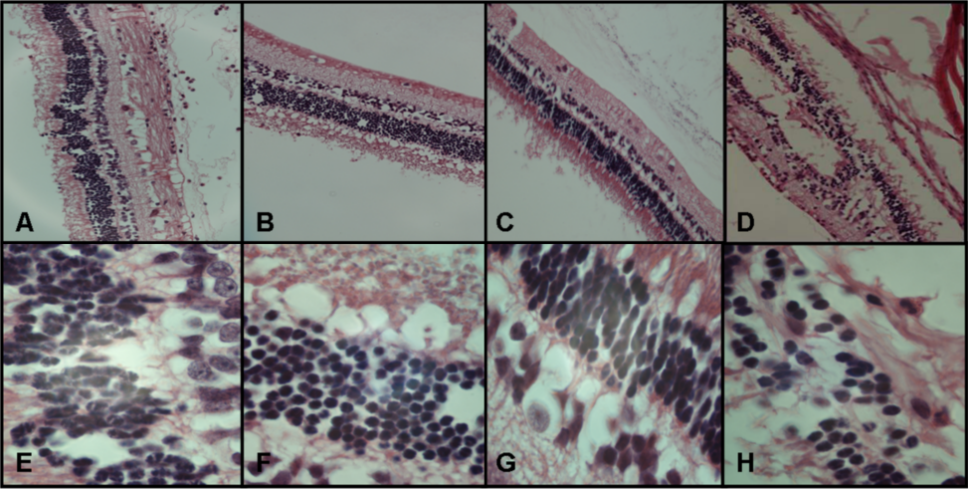
**Representative histology showing the retinas of infected eyes of PPSV23 (A,E), PPSV23/PLY (B,F), PLY (C,G), or mock (D,H) passively immunized rabbits 24 hours PI. A**-**D** are 200x and **E**-**H** are 1000x original magnification.

**Figure 3:**
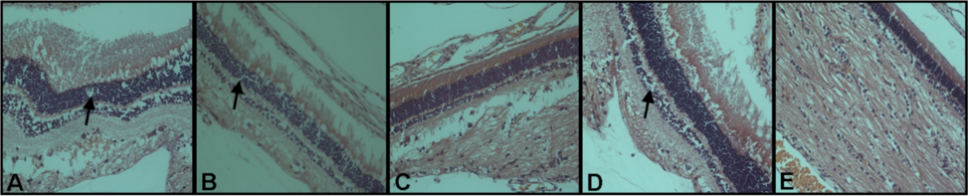
**Representative histology showing the retinas of infected eyes of PPSV23 (A), PPSV23/ΨPLY (B), ΨPLY (C), or mock (D) passively immunized rabbits 24 hours PI treated with vancomycin at 4 hours PI.** (**E**) Representative retina of uninfected eye treated with vancomycin at 4 hours PI. **A**-**E** are 200x original magnification. Black arrows indicate edema.

Less destruction of the retina was observed in infected eyes of PLY, PPSV23 and PPSV23/PLY passively immunized rabbits as compared to mock passively immunized rabbits (Figure 2). The retinas of rabbits passively immunized with PPSV23, PPSV23/PLY, or PLY antibodies retained much of their cellular layers, whereas the control retina appeared to be highly disrupted. When vancomycin was administered 4 hours PI to passively immunized rabbits, only slight pathology, if any, was observed in retinas for all groups (Figure 3). Slightly more edema (black arrows) was observed in the retina of PPSV23, PPSV23/PLY and mock passively (Figure 3A, B, and D; respectively) immunized rabbits as compared to PLY passively immunized rabbits or eyes receiving only vancomycin (Figure 3C and E; respectively).

### MPO activity assay

MPO activity correlates to the amount of neutrophils present in a sample. At 24 hours PI, the infected vitreous of rabbits passively immunized with mock serum had the highest average MPO activity of all passive immunization groups. Significantly lower MPO activity was present in the infected vitreous of rabbits passively immunized with PPSV23/PLY and PLY antisera compared to the vitreous of rabbits that were given mock sera (P = 0.0480). No significant difference was observed among any other group of passively immunized rabbits (P = 0.1165; Table 4). Overall, MPO activity was low or undetectable for all groups receiving passive immunization combined with administration of intravitreal vancomycin (P = 0.3359; Table 5).

**Table 4:** MPO units/mL* recovered from vitreous of passively immunized rabbits

**Passive immunization group**	**24 hours PI**
PPSV23	2.92 ± 1.04
PPSV23/ΨPLY	2.17 ± 0.47^+^
ΨPLY	2.88 ± 0.66^+^
Mock	5.64 ± 1.23

* ± SEM.

^+^Significantly lower than mock passively immunized rabbits.

N = 7 eyes per group.

**Table 5:** MPO units/mL* recovered from vitreous of passively immunized rabbits administered vancomycin 4 hours post-infection

**Passive immunization group**	**24 hours PI**
PPSV23	0.00 ± 0.00
PPSV23/ΨPLY	0.09 ± 0.09
ΨPLY	0.00 ± 0.00
Mock	1.33 ± 1.33

* ± SEM.

N = 4 eyes per group.

### Vitreal IgG

Anti-PLY IgG was detected in undiluted vitreous of rabbits passively immunized with PLY or PPSV23/PLY antiserum. Anti-PPSV23 IgG was not detected in the vitreous of rabbits passively immunized with PPSV23 or PPSV23/PLY antiserum. Neither anti-PLY IgG nor anti-PPSV23 IgG was detected in the vitreous of mock passively immunized rabbits (data not shown).

### ERG

A significantly lower percent loss of retinal function was observed in rabbits passively immunized with PLY or PPSV23/PLY antiserum as compared to mock passively immunized rabbits at 24 hours PI (P = 0.0323). Passive immunization with PPSV23 antiserum lowered the percent loss of retinal function when compared to mock immunized rabbits at 24 hours PI, although not significantly (P = 0.1309). No significant difference was observed among any other treatment groups (P = 0.3163; Table 1). A significantly lower percent loss of retinal function was observed for rabbits passively immunized with PPSV23 and PPSV23/PLY compared to mock passively immunized rabbits when vancomycin was administered 4 hours PI (P = 0.0200; Table 2). In the same treatment group, a lower percent loss of retinal function was observed for rabbits passively immunized with PLY antiserum, though not significantly lower than mock passively immunized rabbits (P = 0.4000). Vancomycin treatment alone caused a small, but not significant, percent loss of retinal function.

## Discussion

Currently, Pneumovax^®^ 23 is used for protection against pneumonia in adults. The effectiveness of this vaccine against pneumococcal endophthalmitis has never been reported. A recent study showed that the pneumococcal capsule is a virulence factor for endophthalmitis [21]. Since pneumococcal endophthalmitis can result in severe complications such as loss of vision or ocular morbidity [5-7], the possible protective value of the currently available capsule-based vaccine was investigated. Additionally, PLY has not only been shown to be an important virulence factor for *S. pneumoniae* endophthalmitis [26,27], but has also been shown to be effective as an immunogen for the active immunization of rabbits and subsequent vitreal challenge with *S. pneumoniae*[28]. Therefore, the possibility of using a combination of antisera to both capsule and PLY was investigated to determine whether additive or enhanced protection could be achieved by passive administration.

For the current study, a capsule type 19 F strain was used for infection because capsule type 19 F is included in the polyvalent pneumococcal vaccine. Whereas whole blood from PPSV23 and PPSV23/PLY actively immunized rabbits was able to kill *S. pneumoniae in vitro*, passive administration of antisera from these rabbits did not provide a bacterial clearing effect for the vitreous of the recipient rabbits. We also observed a lack of bacterial clearance in the infected vitreous of the actively immunized animals despite significant protection of the retinas from pathology (data not shown). One explanation could be that complement is involved in the clearing of bacteria. The interior of the eye is an immune privileged area. Far lower amounts of complement proteins are found inside the eye, even when the eye is inflamed due to infection [39,40]. Also, membrane-bound regulatory proteins in the eye block deposition of C3b (deposition involved in opsonophagocytosis of gram-positive bacteria) so that the complement pathway will not destroy self-tissue of the eye. These regulatory complexes are not present in the blood, so components of whole blood were able to opsonize the bacteria [41]. Alternatively, the antibodies administered through passive immunization could have neutralized the binding of bacteria to the tissue and structures inside the eye lowering the inflammatory response.

Another explanation for the differences observed in bacterial recovery *in vivo* compared to bacterial killing *in vitro* is due to disparities in capsule type antibodies. One study showed that the antibody titers to PPSV23 vaccination are not equal for each capsule type [42]. Also, an immortalized cell line producing monoclonal antibody to PPSV23 was shown to only produce antibodies specific to one capsule type included in the vaccine (capsule type 8), but not the 13 other capsule types tested [43]. The capsule type of the bacteria may play a role in how well the bacteria are cleared.

It is of note that less neutrophil activity was observed in the infected vitreous of rabbits passively immunized with PPSV23/PLY or PLY compared to rabbits passively immunized with mock serum. It is not surprising that these immunized animals had higher bacterial loads recovered from the vitreous than mock-immunized animals since fewer neutrophils were present to kill the bacteria. Since anti-PLY IgG but not anti-PPSV23 IgG was detected in the vitreous of the immunized rabbits, it is a likelihood that anti-PLY antibodies may account for the reduction in neutrophil infiltration and thus an increase in bacterial loads.

The lack of bacterial clearance could be circumvented by administration of an antibiotic, which is normally given to patients affected by bacterial endophthalmitis. Vancomycin is a commonly used treatment for gram-positive bacterial endophthalmitis [9,44]. Since administration of passive antibodies was able to protect against retinal damage, we decided to test whether treatment with vancomycin could help clear the bacteria from the vitreous of passively immunized rabbits. In this study, we found that treating the pneumococcal endophthalmitis infection at 4 hours PI, when inflammation was beginning to be observed, completely sterilized the eye. This treatment time was chosen in an attempt to provide some clinical relevance, that is, a time when inflammation was observable and treatment options might be sought. Even with early vancomycin treatment and complete sterility of the eye, immunization was still helpful in significantly lowering the percent loss of retinal function and preserving some of the retinal architecture for rabbits passively immunized with PPSV23 or PPSV23/PLY antiserum.

Immunization strategies to provide some protection against pneumococcal endophthalmitis could be useful in clinical situations. Since ocular surgery, especially cataract surgery, has been documented to be a major cause of bacterial endophthalmitis [5,6,45], and since *S. pneumoniae* is commonly isolated as the cause of post-surgical endophthalmitis [5-7], passive administration of antibodies to capsule and/or PLY in addition to prophylactic or postoperative antibiotics could be attempted to prevent complications. In the same way, those patients that have already been administered a vaccine consisting of capsule and/or PLY for the prevention of invasive pneumococcal diseases may receive additional benefit of protection from retinal damage during endophthalmitis, especially if the treatment includes antibiotics that are effective at sterilizing the eye [46]. These possibilities would need to be examined in humans, however, to determine their plausibility.

## Conclusions

Taken together, the results indicate that passive immunization with PPSV23 and/or PLY is significantly more effective at preserving ocular integrity during pneumococcal endophthalmitis than negative controls. It appears that retinal function fared better in rabbits that were administered PLY or PPSV23/PLY antisera than in those given PPSV23 antisera, although there was some preservation of function in the PPSV23 group. Immunization alone was not able to reduce the bacterial burden in the vitreous, however, the addition of vancomycin provided a synergistic effect of sterilizing the eye while the combination PPSV23/PLY antiserum reduced clinical severity and loss of retinal function.

## Competing interests

The authors declare that they have no competing interests.

## Authors’ contributions

MS and MM conceived and designed the study and contributed in preparation of the study protocol. MS, ST, NT, EN, QM and LK were involved in data collection supervision. MS and HT performed the statistical analyses. MS drafted the manuscript. All authors read and critically revised the manuscript and approved the final draft.

## Pre-publication history

The pre-publication history for this paper can be accessed here:

http://www.biomedcentral.com/1471-2415/13/8/prepub
